# Systematic Review and Meta-Analysis of the Incidence of Rupture, Repair, and Death of Small and Large Abdominal Aortic Aneurysms under Surveillance

**DOI:** 10.3390/jcm12216837

**Published:** 2023-10-29

**Authors:** Nicola Leone, Magdalena Anna Broda, Jonas Peter Eiberg, Timothy Andrew Resch

**Affiliations:** 1 Department of Vascular Surgery, Rigshospitalet, 2200 Copenhagen, Denmark; magdalena.anna.broda.01@regionh.dk (M.A.B.); jonas.peter.eiberg@regionh.dk (J.P.E.); timothy.andrew.resch@regionh.dk (T.A.R.); 2 Department of Vascular Surgery, Ospedale Civile di Baggiovara, Azienda Ospedaliero-Universitaria di Modena, University of Modena and Reggio Emilia, 41126 Modena, Italy; 3 Department of Clinical Medicine, Faculty of Health and Medical Sciences, University of Copenhagen, 1172 København, Denmark; 4 Copenhagen Academy of Medical Education and Simulation (CAMES), 2100 København, Denmark

**Keywords:** aortic pathology, aortic disease, aortic aneurysm, aneurysm, ruptured aneurysm, mortality

## Abstract

Background: The ultimate goal of treating patients with abdominal aortic aneurysms (AAAs) is to repair them when the risk of rupture exceeds the risk of repair. Small AAAs demonstrate a low rupture risk, and recently, large AAAs just above the threshold (5.5–6.0 cm) seem to be at low risk of rupture as well. The present review aims to investigate the outcomes of AAAs under surveillance through a comprehensive systematic review and meta-analysis. Methods: PubMed, Embase, and the Cochrane Central Register were searched (22 March 2022; PROSPERO; #CRD42022316094). The Cochrane and PRISMA statements were respected. Blinded systematic screening of the literature, data extraction, and quality assessment were performed by two authors. Conflicts were resolved by a third author. The meta-analysis of prevalence provided estimated proportions, 95% confidence intervals, and measures of heterogeneity (I^2^). Based on I^2^, the heterogeneity might be negligible (0–40%), moderate (30–60%), substantial (50–90%), and considerable (75–100%). The primary outcome was the incidence of AAA rupture. Secondary outcomes included the rate of small AAAs reaching the threshold for repair, aortic-related mortality, and all-cause mortality. Results: Fourteen publications (25,040 patients) were included in the analysis. The outcome rates of the small AAA group (<55 mm) were 0.3% (95% CI 0.0–1.0; I^2^ = 76.4%) of rupture, 0.6% (95% CI 0.0–1.9; I^2^ = 87.2%) of aortic-related mortality, and 9.6% (95% CI 2.2–21.1; I^2^ = 99.0%) of all-cause mortality. During surveillance, 21.4% (95% CI 9.0–37.2; I^2^ = 99.0%) of the initially small AAAs reached the threshold for repair. The outcome rates of the large AAA group (>55 mm) were 25.7% (95% CI 18.0–34.3; I^2^ = 72.0%) of rupture, 22.1% (95% CI 16.5–28.3; I^2^ = 25.0%) of aortic-related mortality, and 61.8% (95% CI 47.0–75.6; I^2^ = 89.1%) of all-cause mortality. The sensitivity analysis demonstrated a higher rupture rate in studies including <662 subjects, patients with a mean age > 72 years, >17% of female patients, and >44% of current smokers. Conclusion: The rarity of rupture and aortic-related mortality in small AAAs supports the current conservative management of small AAAs. Surveillance seems indicated, as one-fifth reached the threshold for repair. Large aneurysms had a high incidence of rupture and aortic-related mortality. However, these data seem biased by the sparse and heterogeneous literature overrepresented by patients unfit for surgery. Specific rupture risk stratified by age, gender, and fit-for-surgery patients with large AAAs needs to be further investigated.

## 1. Introduction

Ruptured abdominal aortic aneurysms (AAAs) have a mortality rate of approximately 80% [1]. Although prophylactic endovascular aortic repair (EVAR) and open aortic repair (OAR) are valid treatment options, repair is not without risks of mortality and complications [2,3]. The ultimate goal of treating patients with abdominal aortic aneurysms (AAAs) is to repair them when the risk of rupture exceeds the risk of repair.

The association between diameter and rupture risk is well established, and randomized control trials (RCTs) have confirmed that the repair of AAAs smaller than 5.5 cm in maximum diameter should be avoided [4,5,6]. Based on these findings, the current guidelines suggest elective repair when the maximum anteroposterior aortic diameter is ≥5.0/5.5 cm on ultrasound in women and men, or in cases of rapid growth (≥1 cm/year) [7]. However, these recommendations rely on outdated RCTs powered by historical, perhaps overestimated, AAA rupture data [8,9,10]. Furthermore, the RCTs were flawed by underestimating the surgical operative risk (UK SAT) and by using different methodologies for measuring AAA diameter (UK SAT and ADAM) [11,12]. This information is currently transposed into the National Institute for Health and Care Excellence (NICE) guidelines, confirming the absence of robust evidence to support the 5.5 cm threshold for men [13].

There has been a lack of population-based studies in the last two decades. Between 2009 and 2017, the National Health Service AAA Screening Programme (NAAASP) screened more than 18 65-year-old males with small AAAs (30–55 mm) [14]. The three-year cumulative incidence of rupture was approximately 0.6% [14]. According to a retrospective analysis of a large prospectively maintained database, the three-year cumulative incidence of rupture in patients with AAAs measuring 5.5–6.0 cm and 6.1–7.0 cm was 2.2% and 6.0%, respectively [15]. Thus, small AAAs demonstrate a low rupture risk, and much more surprisingly, large AAAs just above the threshold (5.5–6.0 cm) seem to be at low risk of rupture as well.

The risk of rupture has implications for patient counselling, surveillance protocols, and surgical decision-making. However, updated systematic reviews and meta-analyses summarising the modern outcomes of AAA surveillance are lacking. Therefore, this work aimed to perform a comprehensive systematic review of the evidence on AAA rupture risk and the rate of small AAAs reaching the threshold for repair, aortic-related mortality, and all-cause mortality after the year 2000.

## 2. Materials and Methods

The objectives and methodology of this project were prespecified in the International Prospective Register of Systematic Reviews (PROSPERO) under ID #CRD42022316094. This systematic review and meta-analysis was performed according to the Cochrane Collaboration and PRISMA statements [16]. The search was completed on 22 March 2022 in Medline, Embase, and CENTRAL (Cochrane Central Register of Controlled Trials), combining thesaurus and free text terms (untreated, nonoperative, risk, rupture, diameter, threshold, growth, size, fate, natural history, surveillance, screening, follow-up, AAA, and abdominal aortic aneurysm) with standard Boolean operators.

### 2.1. Study Selection and Inclusion Criteria

This systematic review evaluated all the available studies with the following inclusion criteria: (i) both men and women, or a single gender, older than 18 years and being part of all ethnic groups; (ii) with an abdominal aortic aneurysm (AAA) of any size (>30 mm; see the Section 2.3 for details); (iii) under surveillance/screening; (iv) with duplex ultrasound scans (DUS), computed tomography angiography (CTA), or magnetic resonance (MR) imaging; (v) reporting a rupture rate and/or rate of small aneurysms reaching the threshold for repair; (vi) with a follow-up initiated after the year 2000. Interventional or observational and prospective or retrospective study designs were considered eligible.

Meta-analysis and reviews were excluded using the ‘Publication type’ option. Exclusion criteria included: (i) studies not reporting the rate of rupture or the baseline size of the small aneurysm reaching the threshold for repair; (ii) studies reporting on aortic ectasia or on aortic segments other than abdominal; (iii) studies focusing on operative management; and (iv) follow-ups initiated before the year 2000. Authors responsible for either included or excluded papers were not contacted. No language or other constraints were applied.

### 2.2. Data Collection Process and Quality

The literature search result was uploaded and managed through Covidence systematic review Software, Veritas Health Innovation, Melbourne, Australia (available at www.covidence.org), allowing two authors (N.L. and M.A.B.) to perform a blinded systematic screening of the literature search result. A senior author (T.A.R.) resolved disagreements. Each title and abstract were evaluated for inclusion/exclusion criteria. Studies assessed as having an eligible abstract underwent a blinded full-text screening. Finally, the screening authors extracted data from included publications using a data collection form that was established a priori following an internal discussion. A study quality assessment (the Quality Appraisal Checklist from the Institute of Health Economics) [17] was performed simultaneously. For the primary outcome, publication and reporting biases were assessed by evaluating funnel plot asymmetry. Egger’s test was used to evaluate small study effect biases.

### 2.3. Outcomes and Definitions

The primary outcome was the incidence of AAA ruptures during surveillance. Secondary outcomes were (i) the rate of small AAAs reaching the threshold for repair, (ii) aortic-related mortality, and (iii) all-cause mortality.

As suggested by the current guidelines, an AAA was defined as a dilation of ≥30 mm [7]. Aneurysms were classified as small if the diameter ranged between 30 and 55 mm, considering that in this case prophylactic repair is not recommended [7]. Correspondingly, a large AAA was defined as a diameter exceeding 55 mm. The rate of small AAAs reaching the threshold for repair was extracted by the current authors as presented in the literature. Aortic-related mortality accounts for death caused by the aneurysm directly (rupture) or indirectly (e.g., infection). All-cause mortality includes all etiologies leading to death. The thought behind presenting overlapping diameter groups was to evaluate eventual differences between diameter subgroups; e.g., the small AAA group outcomes might be overshadowed by the inclusion of very small aneurysms (<40 mm) in contrast with the 40–55 mm subgroup. The outcomes were aggregated, analysed, and presented according to baseline size ranges when a minimum of three publications were available.

There were no attempts to contact primary authors to better clarify the threshold for repair details (e.g., which guidelines were applied, how many patients were women, treatment of different aneurysm morphology at different thresholds, etc.) nor the causes of aortic-related mortality and all-cause mortality. All variables included in the data collection form have been specified in Table A1.

### 2.4. Data Synthesis and Analysis

The outcomes were gathered as proportions for the quantitative analysis. For instance, the small AAA estimate proportion of rupture was calculated by dividing the number of ruptured AAAs ranging from 30 to 55 mm by the total number of patients in the subgroup. This provided the data for pooling proportions in a meta-analysis of multiple studies. Data presented as median and interquartile range were converted into means and standard deviation, according to Hozo and colleagues [18]. The primary outcome was displayed as a forest plot for the size ranges of interest. The 95% confidence interval (95% CI) was based on the Wilson score. The Freeman-Tukey transformation (double arcsine transformation) was applied to avoid negative proportions in the CI (CI range 0–100%) [19]. The heterogeneity of the included studies was managed using the random-effects model [20]. The heterogeneity coming from different studies was examined by either inspecting the scatter in the data points and the CIs overlap as well as by performing I^2^ statistics [21]. Sensitivity analysis was performed for the primary outcome of the most frequently reported size group (30–55 mm) regarding female gender, smokers, study sample size, and mean age of included patients. The cut-offs for meta-regressions were based on median values. Statistical analysis was performed with STATA 15.1 (StataCorp College Station, TX, USA).

## 3. Results

**Figure 1:**
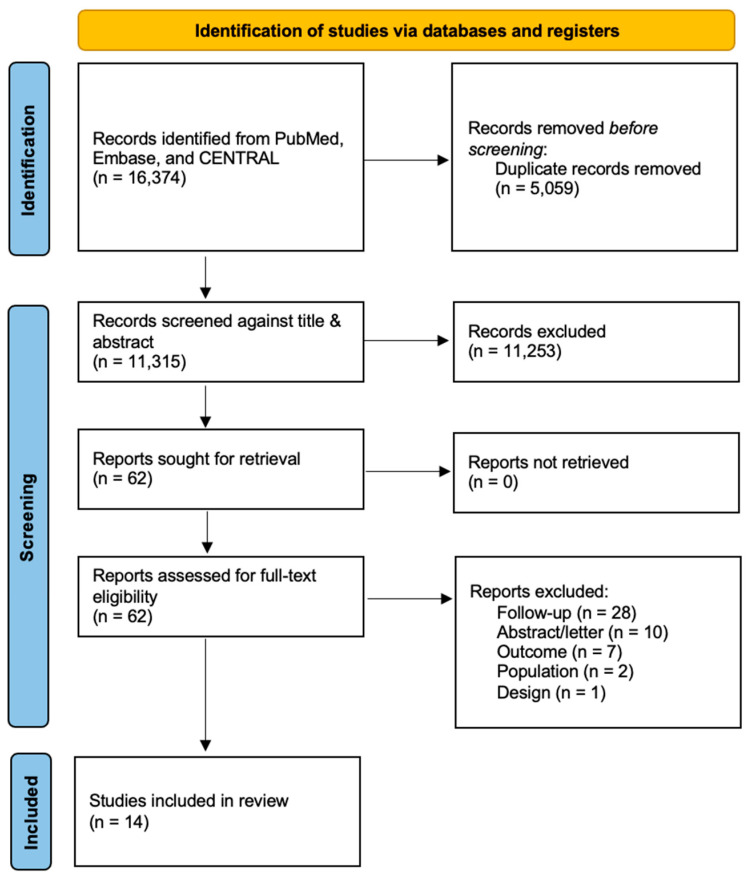
PRISMA flow diagram.

The literature search resulted in 11,315 references after the removal of duplicates (Figure 1).

Of the 62 full texts considered for inclusion, 28 were excluded because the follow-up was initiated before the year 2000; ten were congress abstracts or correspondences; seven did not match the present outcomes of interest; two reported on populations not suitable for inclusion; and one was excluded based on study design (Table A2). Overall, 31,432 participants were reported in the 14 included studies [6,14,15,22,23,24,25,26,27,28,29,30,31,32]. However, the number of patients eligible for analysis in the present meta-analysis was 25,040 due to loss of follow-up (n = 1933), sub-populations not matching the inclusion criteria, and other causes of withdrawal. Nine (64%) publications were European [14,26,28,30,31]; one was a multicenter study including European and western Asian hospitals [6]; and the remaining four publications were from New Zealand (n = 1, 7.2%), Australia (n = 1, 7.2%), the United States of America (n = 1, 7.2%), and Qatar (n = 1, 7.2%) [15,23,25,27]. The baseline and specific details for each included study have been displayed in Table 1 and Table 2.

The quality appraisal is summarised in Table A3. The project has evolved since its initial inception due to the absence of a homogeneous statistical measure of the rupture risk and the heterogeneity of size thresholds reported in the literature. Specifically, a direct comparison of subgroups just below and above the threshold for repair was not possible due to the absence of data.

The pooled estimate of subjects’ mean age was 74.0 years (95% CI 68.7–79.3; I^2^ = 91.7%) [6,14,15,22,23,24,25,26,27,28,29,30,31,32]. The female proportion was 17.4% (95% CI 6.0–32.8; I^2^ = 99.7%) [6,14,15,22,23,24,25,26,27,28,29,30,31,32]. The patients had a mean follow-up of 2.2 years (95% CI 1.4–3.1; I^2^ = 81.6%) [6,15,22,23,24,25,26,27,28,29,31,32]. One study did not report the mean or median follow-up duration [30]. The proportions of current-, previous-, and never-smokers were 44.8% (95% CI 34.0–55.7; I^2^ = 99.2%), 26.2% (95% CI 13.7–41.1; I^2^ = 99.7%), and 11.6% (95% CI 8.7–14.9; I^2^ = 96.1%), respectively [6,14,15,22,24,25,26,27,28,29,32]. However, the sum of the three smoking statuses does not reach 100% because the statuses were heterogeneously reported and different publications were used to estimate the single variable.

### 3.1. Patients with Small AAAs

The overall outcomes of the small aneurysm group (30–55 mm) as well as the mid-sized AAAs (40–55 mm) are shown in Table 3. Seven publications reported on the primary outcome of patients with small aneurysms [6,14,23,24,26,29,32]. One additional study also published the secondary outcomes of small aneurysms [25].

**Figure 2:**
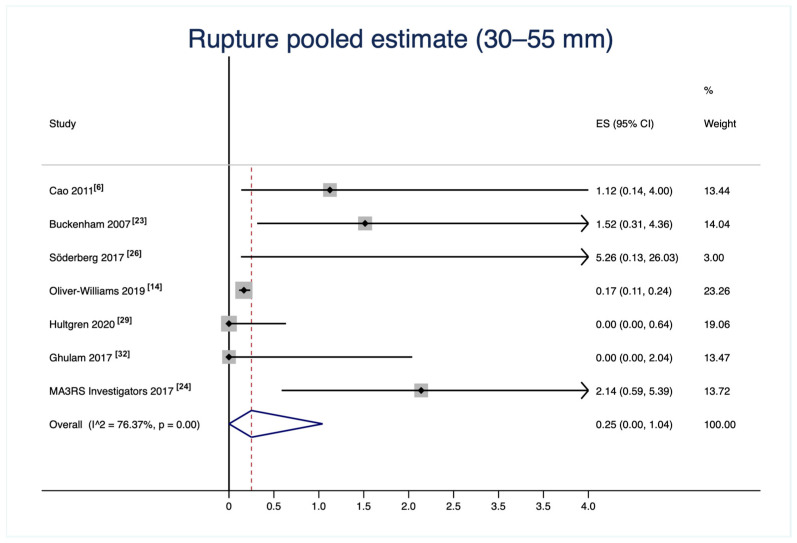
Small abdominal aortic aneurysm pooled estimate of rupture incidence. The vertical dotted line represents the mean proportion of all studies. The black horizontal lines represents the confidence interval of each single study [6,14,23,24,26,29,32].

**Figure 3:**
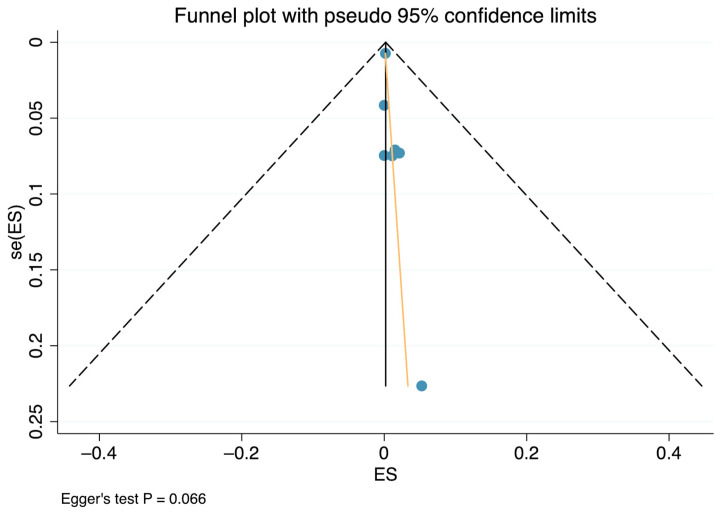
Funnel plot and Egger’s test of small abdominal aortic aneurysm pooled estimate of rupture incidence.

A total of 19,992 small AAAs were analyzed. The incidence of AAA rupture in patients with small AAAs was 0.3% (n = 41 ruptures), with a slight increase in mid-sized AAAs to 0.6% (n = 20 ruptures; subgroup total number of 3498 patients), over a mean follow-up of 2.3 years (95% CI 1.1–3.5; I^2^ = 88.3%). The small AAA group rupture incidence has been graphically illustrated as a forest plot (Figure 2). The corresponding funnel plot demonstrated a fair distribution on average, and Egger’s test *p*-value was higher than 0.05, suggesting the absence of publication biases (Figure 3). The aortic and all-cause deaths were 52 and 1357 vs. 15 and 111 for the 30–55 mm and the 40–55 mm groups, respectively. These data led to 0.6% and 9.6% vs. 0.7% and 5.4% estimated proportions of aortic and all-cause mortality for the 30–55 mm and the 40–55 mm groups, respectively; see Table 3 for details.

The rupture proportion of 30–39 mm AAAs was 0.0% (95% CI 0.0–0.8), and the rate of those reaching the threshold for repair was 1.9% (95% CI 0.9–3.6) during a mean follow-up time of 4.7 years (95% CI 2.6–6.8), according to the single study reporting the subgroup’s outcomes [29]. The rupture proportion in the 30–44 mm subgroup was similar to the one reported for the 30–39 mm subgroup, 0.1% (95% CI 0.1–0.2) [14] within the mean 2.7-year follow-up. The same single publication reported the 30–44 mm subgroup having an aortic-related and all-cause mortality of 0.1% (95% CI 0.1–0.2) and 5.6% (95% CI 5.2–5.9), respectively [14].

All studies reporting the outcomes of 40–49 mm, 45–50 mm, 45–54 mm, and 50–54 mm AAAs were merged under the 40–55 mm size range. Specific outcomes for these groups have been detailed in Table A4.

### 3.2. Patients with Large AAAs

The outcomes of patients with large AAAs (>55 mm) are displayed in Table 3. Four publications reported on rupture, aortic-related mortality, and all-cause mortality of large AAAs [22,28,30,31]. Three studies used a different threshold for large aneurysms > 50 mm, demonstrating a pooled estimate of rupture of 19.0% (95% CI 0.0–60.4; I^2^ = 99.2%) [15,24,29] over a mean follow-up of 2.2 years (95% CI 1.6–2.8; I^2^ = 0.0%). Both primary and secondary outcomes of large AAA subgroups were scarcely reported, leading us to analyse the outcomes of the following sub-groups: 55–60 mm, 61–70 mm, and >70 mm (Table A4). One publication reported the rupture rate for AAAs > 70 mm (57.1%; 95% CI 28.9–82.3) [27] over a mean follow-up of three years. The aortic mortality rate was 41.9% (95% CI 30.5–53.9), 43.9% (95% CI 30.7–57.6), and 43.5% (95% CI 23.2–65.5) for 55–60, 61–70, and >70 mm AAAs, respectively, according to the only publication reporting on this outcome [31]. Primary and secondary outcome data were not available for the remaining size ranges.

### 3.3. Sensitivity Analysis

**Figure 4:**
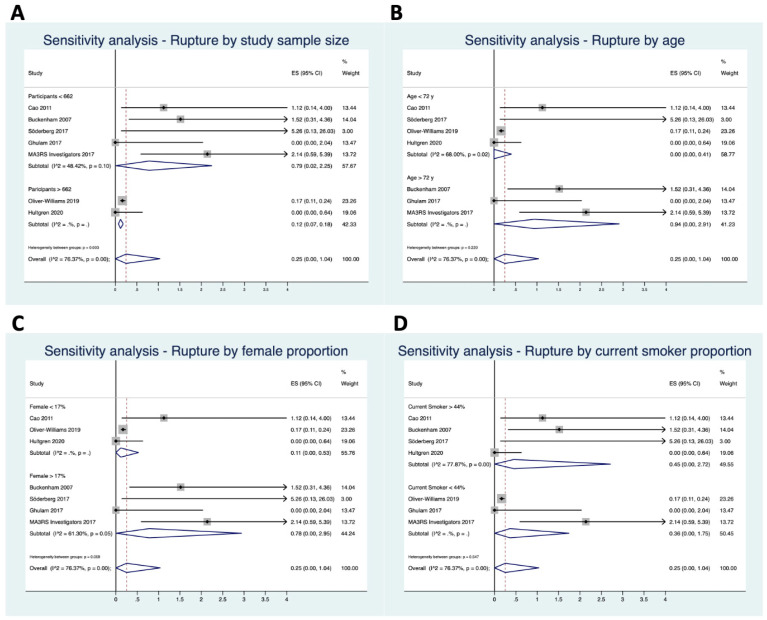
Sensitivity analysis of small abdominal aortic aneurysm pooled estimate rupture (**A**) by sample size (< vs. >662 patients), (**B**) by mean age (< vs. >72 years old), (**C**) by female proportion (< vs. >17%), and (**D**) by current smoker proportion (< vs. >44%). The vertical dotted line represents the mean proportion of all studies. The black horizontal lines represents the confidence interval of each single study [6,14,23,24,26,29,32].

The results of the sensitivity analysis have been graphically depicted in Figure 4.

Overall, the study sample size, mean age, proportion of females, and proportion of current smokers were used for the sensitivity analysis of the seven publications reporting on small aneurysms [6,14,23,24,26,29,32]. The rupture proportion was higher in studies including <662 subjects (0.8% vs. 0.1%; heterogeneity between groups, *p* = 0.003). Furthermore, the rupture proportion was higher within studies including patients with a mean age > 72 years (0.9% vs. 0.0%; heterogeneity between groups, *p* = 0.22). A proportion of female patients exceeding 17% and of current smokers > 44% demonstrated higher estimates of rupture: 0.8% vs. 0.1% (heterogeneity between groups, *p* = 0.059) and 0.5% vs. 0.4% (heterogeneity between groups, *p* = 0.55), respectively.

## 4. Discussion

This meta-analysis confirmed a low incidence of rupture amongst patients with small AAAs (30–55 mm, 0.3%) with a slight increase for patients with mid-sized small AAAs (40–55 mm, 0.6%) over a mean follow-up of 2.3 years. One-fifth (21%) of patients with small AAAs reached the threshold for repair during the same time period. Aortic-related mortality in patients with small AAAs was rare (0.6%), in contrast to all-cause mortality (10%). These results align with the previously published pooled outcomes of small aneurysms, mainly including subjects enrolled before the year 2000 [33]. The rarity of rupture and aortic mortality, as opposed to the non-negligible all-cause mortality, supports non-operative management of small AAAs. The rupture incidence among large aneurysm (>55 mm) patients was 26% over a mean follow-up period of 2.2 years. Of patients with large AAAs, 22% died following an aortic-related complication. The all-cause mortality estimate for this subgroup of patients was 62%. The large AAA rupture rate was higher than the 19% found by Parkinson and colleagues that pooled results of large AAAs turned down from elective repair [34]. We do not have a clear explanation for this; however, several biases and confounders should be considered. First, it is challenging to define the cause of death in patients dying outside healthcare facilities, especially in retrospective studies. Second, the risk of rupture might be exponential, but the pivot point needs further investigation.

Lancaster and colleagues estimated three-year cumulative rupture risks of 2.2%, 6.0%, and 18.4% for AAAs measuring 55–60 mm, 61–70 mm, and >70 mm, respectively [15]. Large population-based screening studies presented a significantly lower rupture rate compared with smaller, observational studies [14,29]. The rupture rates estimated in the present meta-analysis were higher in studies presenting a mean patient age > 72 years (Figure 4B) and with a female proportion > 17% (Figure 4B). The higher risk of rupture associated with ageing seems easily understandable. On the other hand, the higher risk of rupture in those studies, including a relevant number of women, deserves careful discussion. It should be noticed that females with 50–55 mm AAAs seem to be at higher risk of rupture compared with males with 55–60 mm AAAs, 3.4% vs. 2.2%, respectively [15]. However, the 50 mm cut-off for women is debated, and conflicting findings have been found recently [35,36]. Some colleagues strongly support the above-mentioned cut-off as opposed to others proposing a 52 mm threshold [35,36]. To conclude, most published studies support a lower threshold for elective AAA repair in females, eventually meaning that different screening protocols might be required. These results stand in contrast with conducting population screening on 65-year-old men only [14,29].

Thirteen years have passed since Powell et al. [33] published a systematic review on rupture rates of small aneurysms. Still, after all this time, the most relevant finding we can confirm is the scarcity of high-quality evidence investigating the modern fate of AAAs. Nine years have passed since the last systematic review reporting on large aneurysms deemed unfit for elective repair [34]. Our literature search showed more than eleven thousand potential studies, yet the eligibility assessment resulted in a very low number of included studies (n = 14). In addition, the absence of specific reporting standards yields a huge heterogeneity among the papers. We have found twelve different size thresholds, making the pooling process very challenging. Even the definition of a large aneurysm was not uniform, with some studies using a 50 mm cut-off instead of the 55 mm suggested by the guidelines.

The reasoning behind the year 2000 cut-off was the substantial improvement in cardiovascular medical management during the last two decades. A recent meta-analysis confirmed the mortality reduction in AAA patients receiving statins [37]. Metformin treatment showed similar benefits in a prospective study from Australia investigating predictors of AAA outcomes [25]. Stronger evidence is expected to come from an ongoing randomised trial (MAAAGI) [38]. Unfortunately, the extracted publications commonly waived a detailed report of the medications given to the patients, and we were unable to further study this topic.

### 4.1. Limitations

The most relevant limitation in analysing the AAA natural history literature was the lack of homogeneous, high-level, well-powered studies. Assessment of rupture and cause of death is a critical issue, considering that most of the included studies waived the methods employed to ascertain the event’s cause. Hultgren and colleagues pointed out this issue, concluding that the low autopsy rate leads to a ‘difficult and imprecise’ evaluation of the causes of death in such studies [29]. Screening studies focusing on small aneurysms have been performed on relatively young male patients, overshadowing the AAA natural history in the female gender, which has still not been adequately investigated. The rate of repair before reaching the counselled threshold and the number of AAAs not receiving surgery after reaching the threshold were not available. In addition, female-specific size definitions, repair thresholds, and ruptures were commonly not reported, hindering the present authors from providing gender-detailed data. Studies on large aneurysms included patients deemed unfit for surgery, likely biasing the data for fit, less comorbid, and younger patients with large AAAs. Also, there is a lack of data divided by large aneurysm subgroups. An additional limitation is the common avoidance of reporting methods to ascertain the causes of mortality. Similarly, the repair threshold has not been clearly reported, biasing its interpretation. Yet, the pooling process was challenging, with a low number of items per size range likely leading to huge heterogeneity. Also, analysis became more difficult when meta-regressions were performed due to the non-systematic reporting of comorbidities and medical therapies. For this reason, we did not pursue some of the secondary analyses originally planned. As less than ten studies were included in the final analysis, the results should be interpreted with caution. The mean estimated follow-up was short (2.2 years) and stands in contrast to the disease’s natural history. The diagnostic technique varied significantly, and the measuring methodologies (e.g., leading edge to leading edge, inner or outer diameter, etc.) were commonly waived. To conclude, most AAA publications focused on EVAR during the last few decades.

### 4.2. Gaps in Knowledge and Future Perspectives

- Reporting standards defining either the outcomes or the size thresholds are needed.
- Age-stratified rupture risk should be investigated.
- Women deserve gender-focused studies.
- The outcomes of large, fit-for-surgery AAA patients are unknown.
- Repair indications in specific subgroups, such as females and rapid growth, should be further pioneered.
- A new trial using artificial intelligence might improve measuring standardisation, either in the case of computed tomography or duplex ultrasound.

## 5. Conclusions

The rarity of rupture and aortic mortality supports the ongoing guidelines to avoid prophylactic repair of small AAAs (<55 mm). Surveillance of small AAAs seems indicated, considering that one-fifth of patients reach the threshold for repair. The pooled estimate of ruptures and aortic mortality in patients with large aneurysms (≥55 mm) was high, though such crude stratification of size seems unnuanced. There is recent evidence showing that AAAs measuring 55–60 mm in males and 50–55 mm in females might have a reasonably low rupture risk. The modern fate of AAAs is not studied adequately in prospective, controlled trials, and further scientific efforts must be undertaken.

## Figures and Tables

**Table 1:** General information from the fourteen studies included in the meta-analysis.

Author and Publication Year	Country	Journal	Study Period—y	Aim	AAA ^a^	Male|Female ^a^	Age—y ^b^	Follow-Up—y ^b^
Cao 2011 [6]	Europe and Western Asia	EJVES	2004–2008	Surveillance vs. EVAR for small AAAs	178	172|6	68.8	2.6
Buckenham 2007 [23]	New Zealand	NZMJ	2000–2005	Surveillance programme based on the UK SAT	198	148|50	72	1.6
Söderberg 2017 [26]	Sweden	EJVES	2007–2014	5-year natural history of sub-AAAs and AAAs in 70-year-old women	19	0|19	70.0	5.0
Scott 2016 [28]	UK	EJVES	2006–2013	Survival from AAAs not undergoing immediate repair	138	115|23	77.0	2.3
Oliver-Williams 2019 [14]	UK	Circulation	2009–2017	Safety of men under surveillance in NAAASP	18,652	18,652|0	66.8	2.7
Noronen 2013 [31]	Finland	EJVES	2000–2012	Fate of an AAA meeting treatment criteria but not the operative requirements	154	106|48	79.6	1.6
Lim 2015 [30]	UK	EJVES	2001–2013	Examine men from the GASP	59	59|0	71.0	-
Lancaster 2022 [15]	USA	JVS	2003–2020	Impact of large AAA sizes on the incidence of rupture and mortality	3248	2312|936	83.6	3.6
Hultgren 2020 [29]	Sweden	Angiology	2010–2017	Long-term follow-up of men in a population-based regional screening programme	662	662|0	65.0	4.7
Golledge 2019 [25]	Australia	EJVES	2002–2017	Determine whether AAA-related clinical events were lower in patients under metformin	1080	881|199	73.4	2.6
Ghulam 2017 [32]	Denmark	EJVES	2013–2015	Surveillance of small AAAs with a new, non-invasive 3D-US	179	146|33	74.1	1
Elmallah 2018 [22]	Ireland	Vascular	2006–2017	Outcome of conservative management of large AAAs unfit for surgery	76	54|22	80.0	2.1
Al-Thani 2014 [27]	Qatar	Angiology	2004–2008	Outcomes of AAAs incidentally discovered	55	50|11	67.0	3.0
MA3RS Investigators 2017 [24]	UK	Circulation	2012–2014	Determine whether USPIO-enhanced MRI could predict the rate of AAA expansion, rupture, or surgical repair	342	292|50	73.1	2.7

^a^ Data are presented as counts; ^b^ Data are presented as means. AAA, abdominal aortic aneurysm; NZMJ, New Zealand Medical Journal; UK SAT, United Kingdom Small Aneurysm Trial; EJVES, European Journal for Vascular and Endovascular Surgery; NAAASP, National Health Service AAA Screening Programme; JVS, Journal for Vascular Surgery; 3D-US, three-dimensional ultrasound; USPIO, ultrasmall superparamagnetic iron oxide; MRI, magnetic resonance imaging.

**Table 2:** Outcomes data extracted from the 14 studies included in the meta-analysis.

Author and Publication Year	Size Range—mm	AAA ^a^	Rupture ^b^	Threshold for Repair ^b^	Aortic Mortality ^b^	All-Cause Mortality ^b^
Cao 2011 [6]	41–54	178	2 (1.2)	75 (42.1)	1 (0.6)	8 (4.5)
Buckenham 2007 [23]	30–55	198	3 (1.5)	52 (26.3)	5 (2.5)	23 (11.6)
Söderberg 2017 [26]	30–55	19	1 (5.2)	6 (31.6)	1 (5.2)	2 (10.5)
Scott 2016 [28]	>55	138	37 (26.8)	-	37 (26.8)	71 (51.4)
Oliver-Williams 2019 [14]	(i) 30–55 (ii) 30–44 (iii) 45–54 (iv) 50–54	(i) 18,652 (ii) 16,430 (iii) 2222 (iv) 769	(i) 31 (0.2) (ii) 20 (0.1) (iii) 11 (0.5) (iv) 3 (0.4)	(i) 1314 (7.0) (ii) - (iii) - (iv) -	(i) 29 (0.2) (ii) 19 (0.1) (iii) 10 (0.5) (iv) -	(i) 980 (5.3) (ii) 912 (5.6) (iii) 68 (3.1) (iv) 15 (2.0)
Noronen 2013 [31]	(i) >55 (ii) 55–60 (iii) 61–70 (iv) >70	(i) 154 (ii) 74 (iii) 57 (iv) 23	(i) 56 (36.4) (ii) - (iii) - (iv) -	-	(i) - (ii) 31 (41.9) (iii) 25 (43.8) (iv) 10 (43.5)	(i) 120 (77.9) (ii) - (iii) - (iv) -
Lim 2015 [30]	>55	59	10 (16.9)	-	10 (16.9)	30 (50.8)
Lancaster 2022 [15]	>50	3 248	216 (6.7)	-	-	756 (23.3)
Hultgren 2020 [29]	(i) 30–55 (ii) 30–39 (iii) 40–49 (iv) 45–50 (v) >50	(i) 579 (ii) 472 (iii) 107 (iv) 35 (v) 76	(i) 0 (0) (ii) 0 (0) (iii) 0 (0) (iv) 0 (0) (v) 2 (2.6)	(i) 42 (7.3) (ii) 9 (1.9) (iii) 33 (30.8) (iv) 0 (0) (v) -	(i) - (ii) - (iii) - (iv) - (v) 1 (1.3)	(i) - (ii) - (iii) - (iv) - (v) -
Golledge 2019 [25]	30–55	952	-	442 (46.4)	12 (1.3)	321 (33.7)
Ghulam 2017 [32]	30–55	179	0	13 (7.3)	0	3 (1.7)
Elmallah 2018 [22]	>55	76	16 (21.1)	-	15 (19.7)	49 (64.5)
Al-Thani 2014 [27]	>70	14	8 (57.1)	-	-	6 (42.9)
MA3RS Investigators 2017 [24]	(i) 40–49 (ii) >50	(i) 187 (ii) 155	(i) 4 (2.1) (ii) 98 (63.2)	(i) 38 (20.3) (ii) -	(i) 4 (2.1) (ii) 13 (8.4)	(i) 20 (10.7) (ii) 28 (18.1)

^a^ Data are presented as counts; ^b^ Data are presented as counts and percentages calculated on the included number of AAAs per specific size range. AAA, abdominal aortic aneurysm.

**Table 3:** Outcomes of pooled estimates for the major size ranges.

	Rupture ^a^	Repair Threshold ^a^	Aortic Mortality ^a^	All-Cause Mortality ^a^
30–55 mm	N = 19,992 e = 41	N = 20,944 e = 1982	N = 20,365 e = 52	N = 20,365 e = 1357
	**0.3**|0.0–1.0|76.4 [6,14,23,24,26,29,32]	**21.4**|9.0–37.2|99.3 [6,14,23,24,25,26,29,32]	**0.6**|0.0–1.9|87.2 [6,14,23,24,25,26,32]	**9.6**|2.2–21.1|99.0 [6,14,23,24,25,26,32]
40–55 mm	N = 3 498 e = 20	N = 507 e = 170	N = 3 356 e = 15	N = 3356 e = 111
	**0.6**|0.1–1.6|57.7 [6,14,24,29]	**33.7**|20.1–48.9|91.9 [6,24,29]	**0.7**|0.0–2.1|71.1 [6,14,24]	**5.4**|1.7–10.9|90.6 [6,14,24]
>55 mm	N = 427 e = 119	-	N = 273 e = 62	N = 427 e = 270
	**25.7**|18.0–34.3| 72.0 [22,28,30,31]	-	**22.1**|16.5–28.3|25.0 [22,28,30]	**61.8**|47.0–75.6|89.1 [22,28,30,31]

N, population available for the specific outcome; e, number of events; ES%, estimate proportion; 95% CI, 95% confidence interval. ^a^ Data are presented as ES%|95% CI|I^2^.

## Data Availability

Data are available on a reasonable request to the corresponding author.

## Appendix A

**Table A1:** Variables extracted from included studies.

	Variables	Type
General	First author name and year of publication	String
	Title	String
	Journal	String
	Study aim	String
	Country	String
	European	Binary
	Study period	String
Methods	Population description (small aneurysms under surveillance, large aneurysms deemed unfit for surgery, aneurysms under surveillance)	Categorical
	Design (RCT, observational, other)	Categorical
	Prospective	Binary
	Multicenter	Binary
	Inclusion criteria	String
	Exclusion criteria	String
	Type of imaging	Categorical
Quality appraisal	Consecutive recruitment	Categorical
	Completeness of baseline characteristics description	Categorical
	Completeness of inclusion/exclusion statement	Categorical
	Entering the study at a similar point in the disease	Binary
	Clarity of intervention/outcome description	Categorical
	Clarity of co-intervention/secondary outcome description	Categorical
	Outcome measures established a priori	Categorical
	Outcome assessors blinded to intervention	Categorical
	Outcomes measured using appropriate objective/subjective methods	Categorical
	Measures made before and after the intervention—Multiple measures over time	Categorical
	Appropriateness/completeness of the statistical analysis	Categorical
	Appropriateness of the follow-up length	Categorical
	Report of losses to follow-up	Binary
	Use of random variability estimates	Categorical
	Conclusions supported by results	Categorical
	Declaration of competing interests and sources of support	Binary
Outcomes	Number of participants	Continuous
	Number of losses to follow-up	Continuous
	Male/Female	Continuous
	Mean (standard deviation) or median (IQR range) of: Length of follow-up Age	Continuous
	Smokers, non-smokers, and previous smokers proportion	Continuous
	Specific outcomes collected for each size threshold (numbers): Patients Rupture Reaching the threshold for repair Aortic-related death All-cause death	Continuous

**Table A2:** Excluded studies after full-text screening.

	**Follow-Up Being Initiated Before the Year 2000**
1	Vega de Céniga et al. Analysis of expansion patterns in 4–4.9 cm abdominal aortic aneurysms. Ann Vasc Surg. 2008;22(1):37–44.
2	Vallabhaneni SR. Final follow-up of the Multicentre Aneurysm Screening Study (MASS) randomized trial of abdominal aortic aneurysm screening (Br J Surg 2012; 99: 1649–1656). Br J Surg. 2012;99(12):1656.
3	Lederle et al. Multicentre study of abdominal aortic aneurysm measurement and enlargement. Br J Surg. 2015;102(12):1480–7.
4	Brown PM, Sobolev B, Zelt DT. Selective management of abdominal aortic aneurysms smaller than 5.0 cm in a prospective sizing program with gender-specific analysis. J Vasc Surg. 2003;38(4):762–5.
5	Lederle et al. Rupture rate of large abdominal aortic aneurysms in patients refusing or unfit for elective repair. Journal of the American Medical Association. 2002;287(22):2968–72.
6	Brown LC, Powell JT. Risk factors for aneurysm rupture in patients kept under ultrasound surveillance. UK Small Aneurysm Trial Participants. Annals of surgery. 1999;230(3):289-96; discussion 296-7.
7	Aziz et al. Four-year follow up of patients with untreated abdominal aortic aneurysms. ANZ J Surg. 2004;74(11):935–40.
8	Valentine et al. Watchful waiting in cases of small abdominal aortic aneurysms—Appropriate for all patients? Journal of Vascular Surgery. 2000;32(3):441–50.
9	Tambyraja et al. Non-operative management of high-risk patients with abdominal aortic aneurysm. Eur J Vasc Endovasc Surg. 2003;26(4):401–4.
10	Devaraj et al. Ultrasound surveillance of ectatic abdominal aortas. Ann R Coll Surg Engl. 2008;90(6):477–82.
11	Schlösser et al. Growth predictors and prognosis of small abdominal aortic aneurysms. Journal of Vascular Surgery. 2008;47(6):1127–33.
12	Scott et al. Randomized clinical trial of screening for abdominal aortic aneurysm in women. British Journal of Surgery. 2002;89(3):283–5.
13	Brady et al. Abdominal aortic aneurysm expansion: Risk factors and time intervals for surveillance. Circulation. 2004;110(1):16–21.
14	Vega de Céniga et al. Growth rate and associated factors in small abdominal aortic aneurysms. Eur J Vasc Endovasc Surg. 2006;31(3):231–6.
15	Powell et al. Rupture rates of small abdominal aortic aneurysms: A systematic review of the literature. Journal of Vascular Surgery. 2011;53(1):249.
16	Thompson et al. Growth rates of small abdominal aortic aneurysms correlate with clinical events. Br J Surg. 2010;97(1):37–44.
17	Propranolol for small abdominal aortic aneurysms: results of a randomized trial. J Vasc Surg. 2002;35(1):72–9.
18	Heikkinen et al. The fate of AAA patients referred electively to vascular surgical unit. Scandinavian Journal of Surgery. 2002;91(4):345–52.
19	Veith et al. Conservative observational management with selective delayed repair for large abdominal aortic aneurysms in high risk patients. J Cardiovasc Surg (Torino). 2003;44(3):459–64.
20	Powell et al. The natural history of abdominal aortic aneurysms and their risk of rupture. Acta chirurgica Belgica. 2001;101(1):11-16.
21	Filardo et al. Immediate open repair vs. surveillance in patients with small abdominal aortic aneurysms: survival differences by aneurysm size. Mayo Clinic proceedings. 2013;88(9):910-919.
22	Mosorin et al. The use of statins and fate of small abdominal aortic aneurysms. Interact Cardiovasc Thorac Surg. 2008;7(4):578–81.
23	Brown et al. The risk of rupture in untreated aneurysms: the impact of size, gender, and expansion rate. J Vasc Surg. 2003;37(2):280–4.
24	Kurvers et al. Discontinuous, staccato growth of abdominal aortic aneurysms. J Am Coll Surg. 2004;199(5):709–15.
25	Powell et al. Final 12-year follow-up of surgery versus surveillance in the UK Small Aneurysm Trial. British Journal of Surgery. 2007;94(6):702–8.
26	Powell et al. Long-term outcomes of immediate repair compared with surveillance of small abdominal aortic aneurysms. New England journal of medicine. 2002;346(19):1445-1452.
27	Solberg et al. Increased growth rate of abdominal aortic aneurysms in women. The Tromsø study. Eur J Vasc Endovasc Surg. 2005;29(2):145–9.
28	Ahmad et al. How Quickly Do Asymptomatic Infrarenal Abdominal Aortic Aneurysms Grow and What Factors Affect Aneurysm Growth Rates? Analysis of a Single Centre Surveillance Cohort Database. Eur J Vasc Endovasc Surg. 2017;54(5):597–603.
	**Congress abstracts or correspondences**
1	Chang et al. Natural History of Abdominal Aortic Aneurysm Expansion: Fifteen-Year Analysis of Nearly 15,000 Patients Under Surveillance in a Large, Integrated Health System. Journal of Vascular Surgery. 2020;72(1):e74–5.
2	Duncan et al. The Subaneursymal Aorta â€“ A Ten Year Perspective from a Single Centre. European journal of vascular and endovascular surgery. 2019;58(6):e21-e22.
3	Bogdanovic et al. Semi-automatic measurement of external and luminal diameter predicts the four-year prognosis of small abdominal aortic aneurysms. Arteriosclerosis, Thrombosis, and Vascular Biology. 2018;38.
4	Lee et al. Growth Rates of Small Abdominal Aortic Aneurysms Identified in a Contemporary Practice. Journal of Vascular Surgery. 2020;72(3):e321–2.
5	Haveman et al. Multicentre Aneurysm Screening S. Multicentre Aneurysm Screening Study (MASS). In: Lancet. England; 2003. p. 1058.
6	Vega de Ceniga et al. Outcomes in a Prospective Cohort of Octogenarian and Nonagenarian Patients Diagnosed With a Small (<55 Mm) Abdominal Aortic Aneurysm: Rupture, Growth to a Large (>=55 Mm) Size and Mortality Rates. European Journal of Vascular and Endovascular Surgery. 2019;58(6):e603.
7	Clarke et al. Turndown for Abdominal Aortic Aneurysm Intervention: A Five Year Follow Up Study. European Journal of Vascular and Endovascular Surgery. 2020;60(2):e57.
8	Lancaster et al. The Natural History of Large Abdominal Aortic Aneurysms in Patients Without Timely Repair: Implications for Rupture and Mortality. Journal of Vascular Surgery. 2020;72(3):e315.
9	Berntsen et al. Familial Abdominal Aortic Aneurysms Don’t Occur Earlier in Life, Neither do they Progress More Rapidly—Observations from Two Population Based Screening Trials. European Journal of Vascular and Endovascular Surgery. 2019;58(6):e555.
10	Brunner-Ziegler et al. Longterm evaluation on the impact of thrombus formation on the course of abdominal aortic diameter expansion. Vasa—Journal of Vascular Diseases. 2013;42:14–5.
	**Not reporting outcomes of interest**
1	Kristensen et al. Glycated Hemoglobin Is Associated With the Growth Rate of Abdominal Aortic Aneurysms: a Substudy From the VIVA (Viborg Vascular) Randomized Screening Trial. Arteriosclerosis, thrombosis, and vascular biology. 2017;37(4):730-736.
2	da Silva et al. The similarities and differences among patients with abdominal aortic aneurysms referred to a tertiary hospital and found at necropsy. Vascular. 2015;23(4):411–8.
3	Badger et al. Surveillance strategies according to the rate of growth of small abdominal aortic aneurysms. Vasc Med. 2011;16(6):415–21.
4	Itoga et al. Metformin prescription status and abdominal aortic aneurysm disease progression in the U.S. veteran patient population. Journal of Vascular Surgery. 2018;67(6):e52.
5	Yau et al. Surveillance of small aortic aneurysms does not alter anatomic suitability for endovascular repair. J Vasc Surg. 2007;45(1):96–100.
6	Lindholt et al. Survival, prevalence, progression and repair of abdominal aortic aneurysms: Results from three randomised controlled screening trials over three decades. Clinical Epidemiology. 2020;12:95–103.
7	Golledge et al. Association between metformin prescription and growth rates of abdominal aortic aneurysms. Br J Surg. 2017;104(11):1486–93.
	**Populations not suitable for inclusion**
1	Hansen et al. Natural history of thoraco-abdominal aneurysm in high-risk patients. Eur J Vasc Endovasc Surg. 2010;39(3):266–70.
2	Chun et al. Risk of developing an abdominal aortic aneurysm after ectatic aorta detection from initial screening. J Vasc Surg. 2020;71(6):1913–9.
	**Study design**
1	Skibba et al. Reconsidering gender relative to risk of rupture in the contemporary management of abdominal aortic aneurysms. J Vasc Surg. 2015;62(6):1429–36.

**Table A3:** Study quality analysis according to the Quality Appraisal Checklist from the Institute of Health Economics.

Study Objective		
**1.**	Was the hypothesis/aim/objective of the study clearly stated?	**Yes** [6,14,15,22,23,24,25,26,27,28,29,30,31,32] **Partial** **No**
**Study design**		
**2.**	Was the study conducted prospectively?	**Yes** [6,14,15,22,23,24,25,26,29,30,32] **Unclear** **No** [27,28,31]
**3.**	Were the cases collected in more than one centre?	**Yes** [6,14,15,24,25,26,29] **Unclear** **No** [22,23,27,28,30,31,32]
**4.**	Were patients recruited consecutively?	**Yes** [24,27,29,30,31,32] **Unclear** [6,14,15,22,23,25,26,28] **No**
**Study population**		
**5.**	Were the characteristics of the patients included in the study described?	**Yes** [6,15,22,23,24,25,26,27,28,29,30,32] **Partial** [14,31] **No**
**6.**	Were the eligibility criteria (i.e., inclusion and exclusion criteria) for entry into the study clearly stated?	**Yes** [6,15,22,23,24,25,26,27,30,31,32] **Partial** [14,28,29] **No**
**7.**	Did patients enter the study at a similar point in the disease?	**Yes** [6,14,15,22,24,25,27,28,29,30,31,32] **Unclear** **No** [23,26]
**Intervention and co-intervention**		
**8.**	Was the intervention of interest clearly described?	**Yes** [6,14,23,24,25,26,29,30,32] **Partial** [15,22,31] **No** [27,28]
**9.**	Were additional interventions (co-interventions) clearly described?	**Yes** [6,14,15,23,24,25,26,28,29,30,32] **Partial** **No** [27,31]
**Outcome measure**		
**10.**	Were relevant outcome measures established a priori?	**Yes** [6,14,15,23,24,25,26,27,28,29,30,32] **Partial** [31] **No**
**11.**	Were outcome assessors blinded to the intervention that patients received?	**Yes** [14,15] **Unclear** **No** [6,23,24,25,26,27,28,29,30,31,32]
**12.**	Were the relevant outcomes measured using appropriate objective/subjective methods?	**Yes** [14,23,25,26,29,32] **Unclear** [6,15,24,27,28] **No** [22,30,31]
**13.**	Were the relevant outcome measures made before and after the intervention?	**Yes** [6,14,23,24,25,26,29,30,31,32] **Unclear** [15,22,27,28] **No**
**Statistical analysis**		
**14.**	Were the statistical tests used to assess the relevant outcomes appropriate?	**Yes** [6,14,15,22,23,24,25,26,27,29,30,31,32] **Unclear** [28] **No**
**Results and conclusions**		
**15.**	Was follow-up long enough for important events and outcomes to occur?	**Yes** [6,15,24,25,26,28] **Unclear** [14,27,29,30] **No** [23,31,32]
**16.**	Were losses to follow-up reported?	**Yes** [6,14,15,23,24,25,26,28,29,30,31,32] **Unclear** [27] **No** [22]
**17.**	Did the study provided estimates of random variability in the data analysis of relevant outcomes?	**Yes** [6,14,15,24,26,30] **Partial** **No** [22,23,25,27,28,29,31,32]
**18.**	Were the adverse events reported?	**Yes** [6,14,15,22,24,26,27,28,29,30,31,32] **Partial** [23,25] **No**
**19.**	Were the conclusions of the study supported by results?	**Yes** [14,15,22,24,25,26,30,31,32] **Unclear** [23,27,28,29] **No** [6]
**Competing interests and sources of support**		
**20.**	Were both competing interests and sources of support for the study reported?	**Yes** [6,14,15,22,24,25,26,27,28,29,31,32] **Partial** **No** [23,30]

**Table A4:** Outcomes pooled estimates for the minor size ranges of small and large aneurysms.

	Rupture ^a^	Repair Threshold ^a^	Aortic Mortality ^a^	All-Cause mortality ^a^
**40–49 mm**	n = 294 e = 4	n = 294 e = 71	n = 187 e = 4	n = 187 e = 20
	1.0 (0.1–2.6) [24,29]	24.0 (19.2–29.0) [24,29]	2.1 (0.6–5.4) [24]	10.7 (6.7–16.0) [24]
**45–50 mm**	n = 35 e = 0	n = 35 e = 24	-	-
	0.0 (0.0–10.0) [29]	68.6 (50.7–83.1) [29]	-	-
**45–54 mm**	n = 2222 e = 11	-	n = 2222 e = 10	n = 2222 e = 68
	0.5 (0.3–0.9) [14]	-	0.5 (0.2–0.8) [14]	3.1 (2.4–3.9) [14]
**50–54 mm**	n = 769 e = 3	-	-	n = 769 e = 15
	0.4 (0.1–1.1) [14]	-	-	2.0 (1.1–3.2) [14]
**55–60 mm**	-	-	n = 74 e = 31	-
	-	-	41.9 (30.5–53.9) [31]	-
**61–70 mm**	-	-	n = 57 e = 25	-
	-	-	43.9 (30.7–57.6) [31]	-
**>70 mm**	n = 14 e = 8	-	n = 23 e = 10	n = 14 e = 6
	57.1 (28.9–82.3) [27]	-	43.5 (23.2–65.5) [31]	42.9 (17.7–71.1) [27]

n, population available for the specific outcome; e, number of events; ES%, estimate proportion; 95% CI, 95% confidence interval. ^a^ Data are presented as ES% (95% CI).
